# Sleep Disturbances, Degree of Disability and the Quality of Life in Multiple Sclerosis Patients

**DOI:** 10.3390/ijerph19063271

**Published:** 2022-03-10

**Authors:** Aleksandra Kołtuniuk, Magdalena Kazimierska-Zając, Dominika Pogłódek, Justyna Chojdak-Łukasiewicz

**Affiliations:** 1 Department of Nursing and Obstetrics, Wroclaw Medical University, 51-618 Wroclaw, Poland; dominikapx@gmail.com; 2 Department of Health Humanities and Social Science, Wroclaw Medical University, 50-345 Wroclaw, Poland; magdalena.kazimierska-zajac@umw.edu.pl; 3 Department of Neurology, Wroclaw Medical University, 50-556 Wroclaw, Poland; justyna.chojdak-lukasiewicz@umw.edu.pl

**Keywords:** multiple sclerosis, quality of life, insomnia, daytime sleepiness

## Abstract

Sleep disturbances are pervasive in patients with multiple sclerosis (MS), with incidence about four times higher compared to the general population. The most frequent primary sleep problems include insomnia, restless leg syndrome, sleep-related movement disorders, and sleep-disordered breathing. This study aims to assess the relationships between sleeping problems and the quality of life (QoL) in MS patients. This cross-sectional study was conducted among 152 MS patients (mean age: 36.27 ± 9.60) between November 2018 and February 2019 at a neurological health center in Wroclaw, Poland. The study was based on a questionnaire that included questions concerning sociodemographic and clinical data in addition to the following standardized questionnaires: Athens Insomnia Scale (AIS), Epworth Sleepiness Scale (ESS), and Multiple Sclerosis International Quality of Life (MusiQoL). The degree of physical disability was evaluated following the Expanded Disability Status Scale (EDSS). Analysis of the research material showed that 66.45% of MS patients had insomnia, and 41.45% presented with daytime sleepiness. The QoL of respondents was assessed as average (50.73). Univariate linear regression model analysis showed the effects of professional status, daytime sleepiness, insomnia, and degree of disability on the QoL of MS patients. Sleep disturbances are widespread in MS patients. The presence of sleep disturbances (insomnia and daytime sleepiness) significantly affects the QoL of MS patients.

## 1. Introduction

Multiple sclerosis (MS) is an autoimmune, demyelinating disease of the central nervous system. It is the most common cause of disability in middle-aged adults [1,2]. The worldwide incidence is estimated at 2.8 million people (35.9 per 100,000 population). The global prevalence continues to increase in all regions of the world [3]. Multiple sclerosis usually develops between the age of 20 and 50 and is more frequently diagnosed in women [4]. MS is characterized by various neurological symptoms, depending on which part of the nervous system is affected. Most common symptoms include sensory problems, walking difficulties, retrobulbar neuritis, diplopia, dizziness, and weakness [5].

Appropriate sleep regularity, duration and absence of sleep disturbances are important for healthy sleep and good quality of life (QoL). Sleep duration varies from one person to another and across the lifespan. According to the recommendation of the National Sleep Foundation, adults need 7–9 h of sleep to maintain optimal physical and mental health [6]. Sleep is essential for the regenerative processes of the body and the brain. Homeostatic synaptic downscaling during sleep supports the memory process. Furthermore, adequate sleep plays a role in oligodendrocyte functions, including myelination and proliferation of new immature oligodendrocytes [7]. Meta-regression analyses found statistically significant linear associations between sleep duration at less than six hours and an increase in mortality [8]. In addition, statistically significant linear associations between longer sleep duration and increased mortality and incident cardiovascular disease have been observed [9].

Studies suggest that sleep disturbances are observed four times more frequently in MS patients compared to the general population. The estimated prevalence ranged from 25 to 62%, with a higher prevalence in women [10,11,12]. Sleep disturbances are associated with an increased risk of cardiac disease, obesity, diabetes and mortality [13]. Sleep disturbances can contribute to pain, depression and fatigue, which are commonly observed in patients with MS, and are associated with stress, psychological strain and suffering, as well as impairment in social, occupational, and other important areas of functioning. Sleep disturbances in MS may affect the efficiency of the remyelination process [14], and they impair cognitive functions, especially attention, working memory, visual memory, verbal memory, and processing speed [15]. The patients with sleep disturbances also reported higher levels of subjective cognitive problems compared to patients with normal sleep [16].

The most frequent sleep disturbances include insomnia, restless leg syndrome (RLS), and obstructive sleep apnea [17]. The sleep disturbances also can be triggered by MS-related symptoms, disease severity, comorbidities, and side effects of the therapy [17]. The primary symptoms of MS that can affect sleep quality are dysfunction of the bladder sphincters, spasticity, neuropathic pain, anxiety, and depression [17,18]. Sleep may also be affected by drugs used in the therapy of MS. Insomnia and decreased REM sleep might be side effects of the treatment of relapses with high doses of steroids [19,20]. Reports on the influence of disease-modifying therapy (DMT) on sleep are ambiguous. Some studies suggest a significant association between poor sleep and immunotherapy, especially that with IFN-beta and glatiramer acetate [21,22,23,24]. IFN-beta treatment was reported to cause hypersomnolence, insomnia, and fatigue. Glatiramer acetate was related to more frequent awakenings and daytime somnolence. However, this association was not found in another study [25]. Drugs used to treat symptoms during MS therapy could potentially influence sleep [24].

The most common sleep problem in people with MS is insomnia, which is defined as an inadequate quantity or quality of sleep associated with one (or more) of the following symptoms: difficulty initiating sleep, difficulty maintaining sleep, or early-morning awakening with inability to return to sleep. According to the available reports, insomnia affects at least 30–40% of MS patients [26]. Lunde et al. [27] reported that patients with MS and depression have insomnia more often. Some studies reported that only 12.5% of MS patients without depression have insomnia [28]. Insomnia is more prevalent in older patients with MS [29]. Insomnia can be episodic or chronic. Episodic insomnia is defined as difficulty in sleeping that lasts a few days, and it is related to the stressor factor [30]. In the case of chronic insomnia, the difficulty in sleeping and the early morning awakening persists for more than three nights a week, and the symptoms last for at least three months [31]. The symptoms of MS, especially pain, neurogenic bladder, and spasticity, can condition the onset of insomnia. Insomnia has a negative impact on QoL [32]. Insomnia and reduced sleep quality are related to fatigue during the day, exhaustion, excessive daytime sleepiness, decreased concentration, impaired daytime functioning, and problems with cognitive impairment [33].

Sleep disturbances are related to sleepiness during the day. Sleepiness is characterized by difficulties staying awake and alert during the day [34]. Sleepiness is interrelated with fatigue and can converge in some situations. Excessive daytime sleepiness can lead to poor attention memory and mood disturbances.

Multiple sclerosis significantly affects the quality of life (QoL) of patients compared to the general population and those with chronic conditions such as diabetes mellitus [35,36]. Furthermore, QoL in MS patients is lower than that of patients with other autoimmune conditions such as rheumatoid arthritis or inflammatory bowel disease [37]. The lower QoL interferes with a patient’s ability to work, pursue leisurely activities, and perform daily life tasks. Many factors contributed to impaired QoL. Based on the literature, male MS patients perceive their QoL significantly worse than females, especially in the physical and social areas [38]. Some studies suggest that a worse QoL can be a significant predictor for the change in the patient’s disability status [39]. Sleep disturbances, mood disturbances, and fatigue often coexist and influence each other and affect QoL [40].

This study aimed to assess the relationships between insomnia, daytime sleepiness, level of disability and the QoL in MS patients.

## 2. Materials and Methods

### 2.1. Participants

A cross-sectional descriptive design with a questionnaire survey was used. The study included 152 MS patients (mean age of 36.27 years) treated at a neurological health center in Wroclaw, Poland. Data were collected between November 2018 and February 2019. The study follows the Strengthening the Reporting of Observational Studies in Epidemiology (STROBE) recommendations.

The inclusion criteria were as follows: (1) confirmed diagnosis of MS based on medical records, (2) stable MS without any episodes of relapse within 30 days before the study, (3) being over 18, and (4) written informed consent to participate in the study. The exclusion criteria were as follows: (1) participants without confirmed MS diagnosis, (2) participants with confirmed MS diagnosis but undergoing treatment for sleep disturbances, (3) patients unable to follow the test instructions, and (4) lack of written consent to participate in the study.

### 2.2. Ethics

The research project was approved by the Bioethics Committee of Wroclaw Medical University, Poland (approval no. KB–45/2018). Participation in the study was anonymous and voluntary. All patients were informed about the study, and their written consent was required to participate in it. The study was carried out following the Declaration of Helsinki and Good Clinical Practice guidelines [41].

### 2.3. Procedure

The respondents were qualified to participate in this study based on the inclusion and exclusion criteria during each check-up visit to the neurological center. During the visit, the neurologist assessed the functional status of the patient using the EDSS scale [42,43]. Afterward, they received traditional pencil-and-paper questionnaires designed to be filled out in approximately 20 min. Researchers also gained access to the full medical records of the patients. In total, 152 surveys were returned.

### 2.4. Measures

The study employed the diagnostic survey method along with the authors’ questionnaire, as well as the following standardized questionnaires: Athens Insomnia Scale (AIS) [44], Epworth Sleepiness Scale (ESS) [45,46], Multiple Sclerosis International Quality of Life (MusiQoL) [47].

#### 2.4.1. Authors’ Questionnaire

This tool was designed by the authors and comprised questions concerning sociodemographic data (i.e., sex, age, place of residence, education, professional activity, and material status) and clinical data (i.e., time from the diagnosis, MS type, type of treatment and complaints).

#### 2.4.2. Athens Insomnia Scale

The AIS is a tool to measure insomnia symptoms based on ICD-10 criteria. It contains eight questions that address issues such as falling asleep, waking up during the night, waking up earlier than scheduled in the morning, total sleep time, sleep quality, mood the next day, physical and mental performance the next day, and daytime sleepiness. Each question is answered on a point scale from 0 to 3, where 0 indicates no difficulty and 3 indicates considerable difficulty. A maximum of 24 points can be scored on AIS, with a minimum of 0. A higher score on this scale indicates poorer sleep quality, and a score of 8 or greater indicates insomnia. Cronbach’s alpha for the Polish version is 0.9 [44].

#### 2.4.3. Epworth Sleepiness Scale

The ESS is a tool used to assess excessive daytime sleepiness. It consists of eight questions that address the likelihood of falling asleep in defined situations, including sitting and reading, watching TV, sitting quietly after dinner without alcohol, sitting and having a conversation, and sitting in a car for a few minutes of stopping in traffic or at a red light. A scale from 0 to 3 is provided for each question as an answer, where 0 is zero probability of falling asleep, 1—low, 2—average, and 3 is high probability of falling asleep. A maximum of 24 points can be scored on the questionnaire. A score below 10 indicates the absence of excessive sleepiness, while a score above 14 indicates pathological sleepiness [45]. Cronbach’s alpha for the Polish version is 0.82 [46].

#### 2.4.4. Multiple Sclerosis International Quality of Life

The MusiQoL is an international QoL questionnaire for patients with multiple sclerosis. It concerns the patient’s living standard in the past four weeks. It contains 31 questions concerning symptoms. They describe nine domains: activity of daily living (ADL—8 questions), psychological well-being (PWB—4 questions), symptoms (SPT—3 questions), relationships with friends (RFr—4 questions), relationships with family (RFa—3 questions), relationships with healthcare system (RHCS—3 questions), sentimental and sexual life (SSL—2 questions), coping (COP—2 questions), and rejection (REJ—2 questions). The patient has 6 answers to choose from, where 1 is never/not at all, 2—rarely/somewhat, 3—sometimes/somewhat, 4—often/a lot, 5—always/very much, and 6—not applicable. The total score ranges from 0 to 100 points. The lower the MusiQoL scores, the worse the QoL. The dimensions of the scale exhibited high internal consistency (Cronbach’s alpha from 0.67 to 0.90) [47].

#### 2.4.5. Expanded Disability Status Scale

The EDSS is a method of quantifying disability in MS patients and monitoring changes in their level of disability during the progression of the disease. It consists of 20 points between 0 and 10, which specify intermediate scores between the primary disability levels at intervals of 0.5 points. The scale consists of functional subscales to assess the function of the pyramidal system, cerebellum, brainstem, sensory system, sphincters, sight, higher brain functions; it also includes mobility assessment. The higher the score, the greater the patient’s disability [42,43].

### 2.5. Statistical Analysis

The statistical analysis was performed using Statistica 13 software (TIBCO, Inc., Palo Alto, CA, USA). Arithmetic means, medians, standard deviations, range of variation (extreme values), lower quartile, and upper quartile were calculated for measurable variables. In the case of qualitative variables, their frequency (%) was calculated. Comparisons of qualitative variables were made using the chi-square (χ^2^) test. Comparison of the results of quantitative variables was performed using the Mann–Whitney U test for independent samples or the Kruskal–Wallis test (along with a post-hoc test: Dunn’s test). A correlation analysis was performed using Spearman’s test between questionnaire scores and questionnaire scores and age. In addition, univariate and multivariate analysis of the linear regression model was conducted. A non-standardized and standardized regression coefficient, standard error and the level of statistical significance were determined. The next step was to build a multivariate model (progressive step method). The multivariate model includes variables where *p* ≤ 0.3. An α = 0.05 was used for all comparisons.

## 3. Results

### 3.1. Participants’ Characteristics

The study included 152 participants. The most numerous group were women (80.92%), persons living in cities up to 100 thousand inhabitants (34.21%), persons with secondary education (36.18%), professionally active (64.47%), and rating their material status as a middle class (76.32%).

The majority of the study group had MS for 1 to 5 years (33.55%). The most frequent among the study group was the relapsing-remitting MS (82.25%). In 125 patients (82.24%), immunosuppressive or immunomodulatory therapy was applied. Tecfidera was the most common medication among patients receiving the modifying treatment, as it was administered to 38 participants (30.40%). Most respondents complained of fatigability (89.47%). Detailed information about the study group is presented in Table 1.

### 3.2. AIS Score and the Relationship between Sociodemographic Data, Clinical Data and Complaints

Analysis of the data obtained through AIS scores showed that 51 patients (33.55%) had no insomnia, while 101 (66.45%) suffered from insomnia.

It was also shown that none of the analyzed sociodemographic data were statistically associated with the frequency of insomnia. Moreover, none of the clinical data were statistically associated with the frequency of insomnia. In contrast, insomnia was found to be significantly more frequently diagnosed in those complaining of sphincter disorders (23.08% vs. 76.92%; *p* = 0.005), fatigability (30.15% vs. 69.85%; *p* = 0.001), sexual disorders (19.23% vs. 80.77%; *p* = 0.007), and mood disorders (21.35% vs. 78.65%; *p* = 0.000).

### 3.3. ESS Score and the Relationship between Sociodemographic Data, Clinical Data and Complaints

Analysis of data obtained through the Epworth Sleepiness Scale showed that pathological sleepiness was present in 12.5% of respondents, moderate sleepiness in 28.95%, and 58.55% of respondents reported no excessive daytime sleepiness.

It was also shown that none of the sociodemographic data, clinical data and complaints statistically significantly differentiated the occurrence of daytime sleepiness (*p* > 0.05).

### 3.4. MusiQoL Score and the Relationship between Sociodemographic Data, Clinical Data and Complaints

The QoL of multiple sclerosis patients was classified as average ($\overset{-}{x}$ = 50.73 ± 10.50). The lowest QoL was observed in the RFa domain ($\overset{-}{x}$ = 31.35 ± 27.50). In contrast, QoL was rated highest in the REJ domain ($\overset{-}{x}$ = 72.33 ± 25.70). Detailed data are shown in Table 2.

Professional status was significantly associated with the QoL (*p* = 0.009). Post–hoc analysis showed that there was a statistically significant difference between the scores obtained by those who were working (52.29 points) and those who were studying (48.07 points) (*p* = 0.011). There were no statistically significant correlations between clinical data and MusiQoL score (*p* > 0.05). In contrast, the MusiQoL score depended on the presence of accompanying symptoms during illness. Lower QoL of MS patients was found to be significantly more in those complaining of mobility and balance disorders ($\overset{-}{x}$ = 48.72 ± 9.90 vs. $\overset{-}{x}$ = 55.09 ± 10.40; *p* < 0.001), sphincter disorders ($\overset{-}{x}$ = 47.27 ± 10.30 vs. $\overset{-}{x}$ = 54.37 ± 9.40; *p* < 0.001), paresthesia ($\overset{-}{x}$ = 47.87 ± 10.30 vs. $\overset{-}{x}$ = 52.64 ± 10.20; *p* = 0.006), dysphagia ($\overset{-}{x}$ = 41.12 ± 11.60 vs. $\overset{-}{x}$= 51.70 ± 9.90; *p* < 0.001), speech disorders ($\overset{-}{x}$ = 44.23 ± 8.00 vs. $\overset{-}{x}$ = 51.39 ± 10.50; *p* = 0.014), and mood disorders ($\overset{-}{x}$ = 48.06 ± 10.40 vs. $\overset{-}{x}$= 54.49 ± 9.4; *p* < 0.001).

### 3.5. EDSS Score and the Relationship between Sociodemographic Data, Clinical Data and Complaints

In the study group, the mean functional status level was 2.57 (SD = 2.10). The degree of disability did not depend on gender, place of residence, or education (*p* > 0.05). On the other hand, statistically significant correlations were observed between disability and professional status (*p* < 0.001) and material status (*p* = 0.001). The post–hoc analysis indicated that learners had better motor skills than unemployed individuals (*p* = 0.033) and retired individuals (*p* = 0.048), and disability pensioners (*p* < 0.001). Respondents describing their material status as low were characterized by greater disability than those with middle (*p* = 0.005) or high (*p* = 0.003) material status. There was also a statistically significant positive correlation between the EDSS score and age (*r* = 0.513, *p* < 0.001), which means that the older the respondent, the higher the degree of disability according to EDSS. It was also demonstrated that the degree of disability did not depend on any of the clinical variables studied (*p* > 0.05). However, there were differences in the level of disability depending on the presence of complaints. A lower level of disability was found to be significantly more frequently diagnosed in MS patients complaining of mobility and balance disorders ($\overset{-}{x}$ = 3.23 ± 2.00 vs. $\overset{-}{x}$ = 1.15 ± 1.50; *p* < 0.001), sphincter disorders ($\overset{-}{x}$ = 3.55 ± 2.10 vs. $\overset{-}{x}$ = 1.54 ± 1.60; *p* < 0.001), hypertonia ($\overset{-}{x}$ = 3.58 ± 2.20 vs. $\overset{-}{x}$ = 1.97 ± 1.80; *p* < 0.001), and sexual disorders ($\overset{-}{x}$ = 3.02 ± 2.00 vs. $\overset{-}{x}$ = 2.34 ± 2.10; *p* = 0.031).

### 3.6. Relationships between AIS, ESS, EDSS and MusiQoL

Analysis of the relationship between the Athens Insomnia Scale and the Expanded Disability Status Scale (EDSS) revealed a correlation between the variables (*r* = 0.201, *p* = 0.013). Participants with insomnia had higher levels of disability. In contrast, there was no relationship between the Athens Insomnia Scale and the Epworth Sleepiness Scale (*p* = 0.286—meaning that the occurrence of insomnia did not depend on the level of daytime sleepiness). There was also no correlation between the Epworth Sleepiness Scale and the Expanded Disability Status Scale (EDSS) score (*p* = 0.211—meaning that the occurrence of daytime sleepiness did not depend on the patient’s degree of disability).

Analysis of the obtained data revealed correlations between MusiQoL and AIS and EDSS (*p* < 0.05). This means that MS patients’ subjective QoL decreases as insomnia scores increase. Patients with a higher degree of disability also rated their QoL lower. However, there was no relationship between MusiQoL and ESS (*p* > 0.05) (Table 3).

Univariate linear regression model analysis showed the effects of professional status, daytime sleepiness, insomnia, and degree of disability on the QoL of MS patients. The multivariate model confirmed the influence of professional status and degree of disability (Table 4).

## 4. Discussion

Poor sleep is more common in MS patients compared to the healthy group [33,40]. MS patients frequently report poor sleep quality—studies show that the percentage of MS patients with poor sleep quality ranges from approximately 40% (38% in Ćarnickova et al. [29], 43% in Garland et al. [48], 44.1% in Vitkova et al. [49]) to approximately 70% (67.1% in Boe Lunde et al. [33], 69.1% in Mosarrezaia et al. [50], and 70.0% in Tabrizi et al. [51]) Some studies suggest that poor sleep is more common in women [33,52]. In contrast, Merlino et al. [40] did not report any differences between genders and poor sleep quality. Numerous studies indicate that poor sleep quality is associated with the degree of disability [53,54], BMI [23], duration of illness [49], number of comorbidities [48,55], levels of physical activity [55], pain [11,33], anxiety [23,48], depression [23] and fatigue [33,55,56].

Patients with sleep disturbances have an increased risk of developing such conditions as heart disease, obesity, and diabetes mellitus [13,57]. Numerous studies indicate that sleep disturbances are strongly associated with fatigue, pain, and depression and often contribute to disability [40,58]. In 2017 Sahraian [59] suggested that sleep disturbance might trigger an acute MS exacerbation.

One of the most commonly observed sleep disturbances in multiple sclerosis patients is insomnia [60]. Our study showed that 66.45% of the study group suffered from insomnia which is consistent with the results of Łabuz–Roszak et al. (59.6%) [61]. Viana et al. [62] reported that 39.3% of their subjects showed insomnia symptoms. In contrast, Brozek et al. [63] and Alhazzanii et al. [28] reported that only about 13% of the subjects had insomnia, which may be related to the lower average age of patients in both groups. In our research, we did not observe the relationship between insomnia, sociodemographic and clinical data. Alhazzani et al. [28] indicated that the prevalence of insomnia among MS patients differed significantly depending on their educational level and antidepressant therapy. In contrast, Łabuz–Roszak et al. [61] showed an association between AIS score and illness duration, gender, and professional activity. This study found that insomnia is significantly more frequently diagnosed in those complaining of sphincter disorders (*p* = 0.005), fatigue (*p* = 0.009), sexual disorders (*p* = 0.007), and mood disorders (*p* < 0.001). Similar findings were also reported by Łabuz–Roszak et al. [61], where a relationship between AIS and fatigue and depression and anxiety was demonstrated.

Excessive daytime sleepiness was found in 41.45% of participants, which is consistent with the findings of Boe Lunde et al. [33]. However, other studies [29,56,61,63,64] indicate a significantly lower proportion of MS patients with daytime sleepiness. Our study showed that the level of daytime sleepiness did not depend on sociodemographic variables, which is consistent with the findings of other researchers [61,64]. Pokryszko–Dragan et al. [64] indicated that patients diagnosed with depression had significantly lower ESS scores than other groups. Conversely, Łabuz–Roszak et al. [61] reported that patients with more severe depressive symptoms experience more daytime sleepiness. However, this study indicated no relationship between mood disorders and daytime sleepiness.

Analysis of the study material showed that the QoL of the respondents was at an average level (50.73), which is consistent with the findings of Jamróz–Wisniewska et al. [47]. However, other studies [65,66,67,68,69] indicate a significantly higher QoL among MS patients as measured by MusiQoL. In our study, the highest self–rated QoL concerned REJ (72.33), which is consistent with the findings of Beiske et al. [69] and Nickel et al. [67]. Fernandez et al. [68] reported that MS patients also rated their QoL high in the REJ domain. In contrast, the lowest QoL was reported regarding RFa (31.35), which is consistent with the findings of Jamróz–Wiśniewska et al. [47]. However, in other studies [67,69], the domains related to performing daily activities and sex life were rated the lowest. Such a low assessment of the overall QoL is puzzling as the study group consisted of relatively young people, with a low degree of disability, treated with immunomodulatory drugs, which allows us to conclude that it is not the functional status and presence of symptoms that significantly affect the assessment of QoL. Low QoL scores in the domain of family relationships may be due to the lack of adequate support received by MS patients from family members. Furthermore, the healthcare system in Poland does not meet the requirements of patients in terms of support activities, as this domain was also rated very low, which was also confirmed by Jamróz–Wisniewska et al. [47]. According to Beiske et al. [69], QoL scores in ADL (*p* < 0.001) and SPT (*p* = 0.032) were significantly lower in unemployed patients. Our study showed that working patients had a higher QoL than students. Nickel et al. [67] and Fernandez et al. [68] also reported that professional status was a QoL predictor in MS patients. In addition, Fernandez et al. [68] also indicated the effect of symptoms, i.e., involuntary body movements, vibration in legs or arms, weakness in limbs, tingling in limbs, visual problems, problems and difficulty concentrating, fatigue and urinary incontinence on the assessment of the QoL of MS patients, which was confirmed in our study, showing that the MusiQoL score depends on the presence of mobility and balance disorders, sphincter disorders, paresthesias, dysphagia, speech disorders, and mood disorders.

A large population-based study conducted in New Zealand found that excessive daytime sleepiness was associated with insomnia [70]. This relationship was not confirmed in our study or Łabuz–Roszak et al. [61], which may be due to the much smaller study groups. Furthermore, Bøe Lunde et al. [33] showed that excessive daytime sleepiness was not significantly associated with poor sleep among MS patients. In contrast, our study indicated an association between insomnia and degree of disability, although previous studies did not show this relationship [61,62]. This is most likely related to the more severe degree of disability in the study group. However, there was no relationship between daytime sleepiness and level of disability, which is consistent with the findings of Pokryszko–Dragan et al. [64]. Vitkova et al. [53] reported that poor sleep quality was significantly associated with greater disability.

This is the first study evaluating the impact of sleep disturbances on the quality of life in patients with MS in the Polish population. Three studies conducted among Polish MS patients have only indicated the occurrence of sleep disturbances i.e., insomnia and daytime sleepiness. The prevalence of sleep disturbances was significantly different (insomnia from 13.20% [63] to 59.80% [61]; daytime sleepiness from 11.00% [63] to 20.50% [61]—it could have been influenced by the average age of the patients and their functional status (the younger and better functioning patients experienced less often sleep disturbances [63]). Other studies [25,51,54,62,71] point to the existence of a relationship between sleep disturbances (assessed using various tools) and a worse QoL in MS patients, which was also confirmed in this study. In fact, univariate linear regression model analysis showed that both the level of daytime sleepiness and the occurrence of insomnia affects the QoL scores of MS patients. Despite this, the multivariate model showed no effect of insomnia and daytime sleepiness on the QoL of patients with MS. The lack of effect may be due to the fact that in this study, the mean age was quite low (36.27 ± 9.60), most patients had good functional status (low EDSS) and were treated with dimethyl fumarate, which has no effect on sleep, and may translate the results and indicate the influence of other variables (not included in the model) on QoL.

### Study Limitations

There are a few limitations of this research. First of all, all research tools used were of the self–report type, so there is always a risk of self–report bias. Secondly, the study group could have been more extensive and randomly selected. In addition, this study did not include a control group.

## 5. Conclusions

Sleep disturbances are a common complaint observed in MS patients. They do not depend on clinical data, i.e., illness duration, illness form, or treatment type, but they do depend on the symptoms which accompany the illness. The presence of sleep disturbances (insomnia and daytime sleepiness) significantly correlates with the QoL of MS patients. Further research on sleep disturbances and QoL in patients with multiple sclerosis appears necessary.

## Figures and Tables

**Table 1:** Characteristics of the study group.

Variable	Value
Age [years], mean ± SD Min-Max 95% CI	36.27 ± 9.60 18–66 34.70–37.80
Sex, n (%) Female Male	123 (80.92) 29 (19.08)
Education, n (%) Basic or vocational education Secondary education Higher education	7 (4.61) 62 (36.18) 83 (59.21)
Place of residence, n (%) Village City > 100 thousand inhabitants 100–500 thousand inhabitants <500 thousand inhabitants	19 (12.50) 52 (34.21) 39 (25.66) 42 (27.63)
Professional activity, n (%) Student Employed Unemployed Disability Retirement pension	11 (7.24) 98 (64.47) 8 (5.26) 32 (21.05) 3 (1.97)
Material status (subjective assessment), n (%) Low Middle High	22 (14.47) 116 (76.32) 14 (9.21)
Clinical type of MS, n (%) PPMS SPMS PRMS RRMS	9 (5.92) 13 (8.55) 5 (3.29) 125 (82.24)
Disease duration (years), n (%) 0–1 2–5 6–10 11–15 16–20 >20	6 (3.95) 51 (33.55) 48 (31.58) 29 (19.08) 11 (7.24) 7 (4.61)
Treatment, n (%) Only for specific MS symptoms Disease–modifying therapies	27 (17.76) 125 (82.24)
Treatments to modify progression, n (%) Gilenia Extavia Copaxone Tecfidera Rebif Betaferon lub Betaseron Avonex Tysabri	12 (9.60) 2 (1.60) 24 (19.20) 38 (30.40) 12 (9.60) 24 (19.20) 8 (6.4)0 5 (4.00)
Complains, n (%) Vision disorders Mobility and balance disorders Sphincter disorders Paresthesia. neuralgia Fatigability Dysphagia Hypertonia Speech disorders Sexual disorders Mood disorders	72 (47.37) 104 (68.42) 78 (51.32) 61 (40.13) 136 (89.47) 14 (9.21) 57 (37.50) 14 (9.21) 52 (34.21) 89 (58.55)

Abbreviations: RRMS, relapsing remitting MS; SPMS, secondary progressive MS; PPMS, primary progressive MS; PRMS, progressive–relapsing MS.

**Table 2:** Assessment of the overall quality of life and the individual MusiQoL domains.

*N* = 152						
	M	Min–Max	Me	Q1	Q3	SD
ADL	56.36	0.00–96.88	59.38	40.63	75.00	23.70
PWB	47.85	6.25–100.00	43.75	25.00	64.58	23.60
RFr	43.01	0.00–100.00	41.67	25.00	58.33	25.10
SPT	61.60	12.50–100.00	62.50	43.75	75.00	19.30
RFa	31.35	0.00–100.00	25.00	8.33	50.00	27.50
RHSC	44.76	0.00–100.00	41.67	25.00	66.67	26.60
SSL	48.69	0.00–100.00	50.00	25.00	62.50	28.60
COP	49.59	0.00–100.00	50.00	25.00	75.00	28.50
REJ	72.33	0.00–100.00	75.00	50.00	100.00	25.70
MusiQoL total score	50.73	15.75–84.20	51.04	45.43	56.83	10.50

Abbreviations: M, mean; SD, standard deviation; Me, median; Min, minimum; Max, maximum; Q1, lower quartile; Q3, upper quartile; QoL, quality of life; ADL, activities of daily living; PWB, psychological well–being; RFr, relationships with friends; SPT, symptoms; RFa, family relationships; RHCS, satisfaction with the healthcare system; SSL, sentimental and sexual life; COP, coping with the disease; REJ, rejection.

**Table 3:** Spearman’s rank correlations between each studied variable.

	Sleep Disturbances		Functional Status
	AIS	ESS	EDSS
	*r* *, *p*	*r* *, *p*	*r* *, *p*
ADL	***r* = −0.378, *p* < 0.001**	*r* = −0.220, *p* = 0.219	***r* = −0.727, *p* < 0.001**
PWB	***r* = −0.481, *p* < 0.001**	*r* = −0.335, *p* = 0.057	*r* = −0.101, *p* = 0.215
RFr	***r* = 0.392, *p* < 0.001**	*r* = 0.113, *p* = 0.532	***r* = 0.197, *p* = 0.016**
SPT	***r* = −0.505, *p* < 0.001**	*r* = −0.332, *p* = 0.059	***r* = −0.163, *p* = 0.044**
RFa	***r* = 0.345, *p* < 0.001**	*r* = −0.061, *p* = 0.735	*r* = 0.127, *p* = 0.123
RHSC	***r* = 0.271, *p* < 0.001**	*r* = 0.278, *p* = 0.117	*r* = 0.099, *p* = 0.226
SSL	***r* = 0.222, *p* = 0.008**	*r* = −0.144, *p* = 0.439	*r* = 0.112, *p* = 0.182
COP	***r* = −0.366, *p* < 0.001**	*r* = −0.107, *p* = 0.552	*r* = −0.071, *p* = 0.386
REJ	***r* = −0.296, *p* < 0.001**	*r* = −0.108, *p* = 0.549	***r* = −0.425, *p* < 0.001**
MusiQoL total score	***r* = −0.194, *p* = 0.016**	*r* = −0.287, *p* = 0.105	***r* = −0.** **208, *p* = 0.** **010**

Notes: * Spearman’s correlation coefficient; in bold—statistically significant correlation (*p* < 0.05). Abbreviations: ADL, activities of daily living; PWB, psychological well–being; RFr, relationships with friends; SPT, symptoms; RFa, family relationships; RHCS, satisfaction with the healthcare system; SSL, sentimental and sexual life; COP, coping with the disease; REJ, rejection; EDSS, Expanded Disability Status Scale; AIS, Athens Insomnia Scale; ESS, Epworth Sleepiness Scale.

**Table 4:** Linear regression analysis.

		MusiQoL–Linear Regression									
		Simple Linear Regression Analysis					Multiple Linear Regression Analysis				
		B	SE	t	*p*-Value	ß	B	SE	t	*p*-Value	ß
Sex	Male	Ref.					–				
	Female	−0.87	1.08	−0.80	0.424	−0.07	–	–	–	–	–
Place of residence	Village	Ref.					–				
	City > 100 thousand inhabitants	2.24	1.36	1.65	0.102	0.14	–	–	–	–	–
	100–500 thousand inhabitants	−2.31	1.49	−1.55	0.123	−0.13	–	–	–	–	–
	<500 thousand inhabitants	1.19	1.47	0.81	0.418	0.07	–	–	–	–	–
Education	Basic or vocational education	Ref.					–				
	Secondary education	−0.28	1.67	−0.17	0.867	−0.01	–	–	–	–	–
	Higher education	0.45	1.59	0.28	0.777	0.03	–	–	–	–	–
Professional activity	Employed	Ref.					Ref.				
	Student	**−6.38**	**2.84**	**−2.25**	**0.026**	**−0.38**	**−9.96**	**2.94**	**−3.39**	**0.001**	**−0.59**
	Unemployed	−0.62	3.19	−0.19	0.846	−0.03	0.08	3.08	0.03	0.979	0.00
	Disability	−0.62	2.09	−0.30	0.766	−0.05	0.66	2.14	0.31	0.757	0.05
	Retirement pension	3.60	4.80	0.75	0.455	0.18	7.17	4.70	1.52	0.130	0.36
Disease duration	0–1	Ref.					–				
	2–5	2.12	1.71	1.24	0.217	0.11	–	–	–	–	–
	6–10	1.00	1.74	0.58	0.564	0.05	–	–	–	–	–
	11–15	2.22	2.01	1.10	0.272	0.10	–	–	–	–	–
	16–20	−2.76	2.87	−0.96	0.337	−0.09	–	–	–	–	–
	>20	−0.46	3.47	−0.13	0.895	−0.01	–	–	–	–	–
ESS	no excessive daytime sleepiness	Ref.					Ref.				
	moderate sleepiness	2.03	1.36	1.49	0.138	0.17	2.52	1.32	1.91	0.058	0.21
	pathological sleepiness	**−3.91**	**1.71**	**−2.29**	**0.024**	**−0.26**	2.52	1.32	1.91	0.058	0.21
Age		0.04	0.09	0.43	0.671	0.03	–	–	–	–	–
EDSS		**−1.14**	**0.40**	**−2.89**	**0.004**	**−0.23**	**−1.33**	**0.47**	**−2.82**	**0.005**	**−0.27**
**AIS**		**−0.41**	**0.17**	**−2.43**	**0.016**	**−0.19**	–	–	–	–	–

Abbreviations: B, unstandardized regression coefficient B; SE, standard error; t, B/standard error; ß, standardized regression coefficient ß., in bold—value statistically significant (*p* < 0.05).

## Data Availability

The authors confirm that all data underlying the findings described in this manuscript is fully available to all interested researchers upon request.
